# 2013 ACC/AHA versus 2004 NECP ATP III Guidelines in the Assignment of Statin Treatment in a Korean Population with Subclinical Coronary Atherosclerosis

**DOI:** 10.1371/journal.pone.0137478

**Published:** 2015-09-15

**Authors:** Chang Hee Jung, Min Jung Lee, Yu Mi Kang, Dong Hyun Yang, Joon-Won Kang, Eun Hee Kim, Duk-Woo Park, Joong-Yeol Park, Hong-Kyu Kim, Woo Je Lee

**Affiliations:** 1 Department of Internal Medicine, Asan Medical Center, University of Ulsan College of Medicine, Seoul, Republic of Korea; 2 Department of Radiology, Asan Medical Center, University of Ulsan College of Medicine, Seoul, Republic of Korea; 3 Department of Health Screening and Promotion Center, Asan Medical Center, University of Ulsan College of Medicine, Seoul, Republic of Korea; Katholieke Universiteit Leuven, BELGIUM

## Abstract

**Background:**

The usefulness of the 2013 ACC/AHA guidelines for the management of blood cholesterol in the Asian population remains controversial. In this study, we investigated whether eligibility for statin therapy determined by the 2013 ACC/AHA guidelines is better aligned with the presence of subclinical coronary atherosclerosis detected by CCTA (coronary computed tomography angiography) compared to the previously recommended 2004 NCEP ATP III guidelines.

**Methods:**

We collected the data from 5,837 asymptomatic subjects who underwent CCTA using MDCT during routine health examinations. Based on risk factor assessment and lipid data, we determined guideline-based eligibility for statin therapy according to the 2013 ACC/AHA and 2004 NCEP ATP III guidelines. We defined the presence and severity of subclinical coronary atherosclerosis detected in CCTA according to the presence of significant coronary artery stenosis (defined as >50% stenosis), plaques, and the degree of coronary calcification.

**Results:**

As compared to the 2004 ATP III guidelines, a significantly higher proportion of subjects with significant coronary stenosis (61.8% vs. 33.8%), plaques (52.3% vs. 24.7%), and higher CACS (CACS >100, 63.6% vs. 26.5%) was assigned to statin therapy using the 2013 ACC/AHA guidelines (*P* < .001 for all variables). The area under the curves of the pooled cohort equation of the new guidelines in detecting significant stenosis, plaques, and higher CACS were significantly higher than those of the Framingham risk calculator.

**Conclusions:**

Compared to the previous ATP III guidelines, the 2013 ACC/AHA guidelines were more sensitive in identifying subjects with subclinical coronary atherosclerosis detected by CCTA in an Asian population.

## Introduction

In November 2013, the American College of Cardiology (ACC) and the American Heart Association (AHA) released new guidelines [1] on the management of blood cholesterol to replace the National Cholesterol Education Program (NCEP) Adult Treatment Panel (ATP) III guidelines updated in 2004 [2]. After the new guidelines released, there have been concerns on increased number of patients eligible for statin treatment [3–5]. In a previous study which used data from the National Health and Nutrition Examination Surveys in the U.S. population, the new guidelines substantially increased the number of adults aged between 60 and 75 years who would be eligible for statin treatment and this effect was mainly driven by an increased number of subjects with a 10-year atherosclerotic cardiovascular disease (ASCVD) risk over 7.5% [4]. In addition, the new guidelines have been reported to potentially overestimate the observed risks of cardiovascular disease (CVD) in primary prevention cohorts [6, 7]. However, debate remains on whether the new guidelines overestimate the risks of CVD, as the missed CVD events, initiation of statins therapy, or revascularization procedure after study enrollment might contribute to the over-estimation of CVD risk in the analyzed cohorts [8, 9]. Furthermore, applying this tool to an Asian population is controversial, as the Pooled Cohort Risk Assessment Equations was developed to predict ASCVD in non-Hispanic Caucasian and African American populations [1, 5].

With the recent development in various imaging modalities for detecting atherosclerosis, screening tests for detecting coronary atherosclerosis are expected to provide important prognostic value on future CVD [10–12]. Among them, the prognostic value of coronary atherosclerosis detected by coronary computed tomography angiography (CCTA) in the assessment of long-term cardiovascular prognosis has been proved in several studies [11, 13, 14].

To date, few studies have compared statin assignment according to the 2013 ACC/AHA guidelines versus the 2004 ATP III guidelines in subjects with coronary atherosclerosis detected by CCTA [15, 16]. However, these previous studies did not adequately reflect primary prevention setting as they included substantial symptomatic patents with chest pain [15, 16]. In addition, a comparative analysis between these two guidelines in terms of subclinical coronary atherosclerosis detected by CCTA has not been performed in Asian populations, which have a much lower incidence of coronary heart disease (CHD) compared to Western populations [17, 18].

In this study, we aimed to investigate whether eligibility for statin therapy as determined by the 2013 ACC/AHA guidelines is better aligned with the presence of subclinical coronary atherosclerosis as detected by CCTA when compared to the 2004 NCEP ATP III recommendations. Furthermore, we tried to determine the optimal cut-off value of the 10-year ASCVD risk for the detection of subclinical coronary atherosclerosis.

## Methods

### Study population

The study population consisted of 7,300 subjects who underwent CCTA using the 64-slice multi-detector computed tomography (MDCT) during routine health evaluations at Asan Medical Center (AMC, Seoul, Republic of Korea) between January 2007 and June 2011. Each subject completed a questionnaire on previous medical and/or surgical diseases, medications, and drinking and smoking habits. Drinking habits were categorized as frequency per week (i.e., ≤1 times/week and ≥2 times/week, moderate drinker); smoking habits as noncurrent or current, and exercise habits as frequency per week (i.e., ≤2 times/week and ≥3 times/week, physically active) [19].

History of CVD was based on physician-diagnosed angina, myocardial infarction, and/or cerebrovascular accidents. Subjects with diabetes were defined as those with fasting plasma glucose (FPG) levels of ≥7.0 mmol/L or hemoglobin A1c (HbA1c) levels ≥6.5% [20]. In addition, subjects who reported the use of anti-diabetic medications on a self-report questionnaire were considered to have diabetes. Hypertension was defined as systolic and/or diastolic blood pressures (BP) ≥140/90 mmHg and/or taking antihypertensive medications. The detailed methods used for clinical and laboratory measurements were described elsewhere [21].

We excluded subjects with a history of CVD (n = 286), as well as those who were on statins (n = 1,158). In addition, subjects that were not between the ages of 20 and 79 years were excluded (n = 19). After exclusion of ineligible subjects, 5,837 subjects without known CVD were enrolled in this study. All subjects provided written informed consent. This study was approved by the Institutional Review Board of the AMC.

### Assignment to statin therapy according to the 2013 ACC/AHA guidelines

Based on the exclusion criteria, no subject included in this study had CVD at the time of enrolment. Subjects who were recommended statin therapy for primary prevention included those with 1) adults ≥21 years of age with primary elevations in low-density lipoprotein cholesterol (LDL–C) ≥190 mg/dL, 2) diabetes who were aged 40 to 75 years with an LDL–C level of 70 to 189 mg/dL, and 3) no clinical ASCVD or diabetes with an LDL–C of 70 to 189 mg/dL and estimated 10-year ASCVD risk of ≥7.5% in individuals aged 40 to 75 years [1]. The 10-year ASCVD risk was estimated using the Pooled Cohort Equations developed by the Risk Assessment Work Group [1]. Parameters required for Pooled Cohort Equations included gender, age, race, total cholesterol, high-density lipoprotein cholesterol (HDL-C), systolic BP, use of antihypertensive medication, diabetes, and smoking [1]. The Pooled Cohort Equations were not applied to subjects younger than 40 years or older than 75 years.

### Assignment to statin therapy according to the 2004 ATP III guidelines

Since we excluded subjects with known CVD, no subject had CHD or stroke at the time of enrolment. Diabetes was regarded as a CHD-risk equivalent [2]. CHD risk factors include smoking, hypertension, low HDL-C (i.e., <40 mg/dL), family history of premature CHD (i.e., CHD in male first-degree relative <55 years of age; CHD in female first-degree relative <65 years of age), and age (men ≥45 years; women ≥55 years) [2]. The 10-year risk for CHD was calculated using the modified Framingham model [22]. Subjects assigned to statin therapy according to the 2004 ATP III guidelines included those categorized as: 1) high-risk (CHD risk equivalents or CHD risk factors ≥2 and 10-year risk for CHD >20%) with an LDL-C level of ≥100 mg/dL, 2) moderately high-risk (CHD risk factors ≥2 and 10-year risk for CHD 10–20%) with an LDL-C level of ≥130 mg/dL, 3) moderate-risk (CHD risk factors ≥2 and 10-year risk for CHD <10%) with an LDL level of ≥160 mg/dL, and 4) lower-risk (0–1 CHD risk factors) with an LDL-C level of ≥190 mg/dL [2].

### MDCT to assess coronary artery stenosis, plaques and calcium score

MDCT examinations were performed by using either 64-slice, single-source computed tomography (CT, LightSpeed VCT; GE, Milwaukee, Wis) or dual-source CT (Somatom Definition or Somatom Definition Flash; Siemens, Erlangen, Germany) as previously described [21]. Stenosis of >50% was defined as significant [23, 24]. Subjects with significant stenosis were further categorized according to the number of diseased vessels; 1-, 2- or 3- vessel disease (VD). 3-VD was defined as either the presence of significant stenosis in all three major epicardial vessels (right coronary, left anterior descending, and left circumflex arteries) or right coronary artery and left main coronary artery disease [25].

Plaques were defined as structures >1 mm^2^ within and/or adjacent to the vessel lumen. Plaques consisting of calcified tissue occupying >50% of the plaque area (density >130 HU in native scans) were classified as calcified (calcified plaque, CAP), plaques with <50% calcium were classified as mixed (mixed plaque, MCAP), and plaques without any calcium were classified as noncalcified lesions (noncalcified plaque, NCAP) [26].

Coronary artery calcium scores (CACS) were measured as described previously [27], and participants were categorized according to the cut-off points used by Greenland et al. (i.e., no, 0; mild, 1 to 100; moderate to severe, 101 to 300; severe >300) [28].

### Statistical analysis

Continuous variables with normal distributions are expressed as the mean ± SD, whereas continuous variables with skewed distributions are expressed as the median (and interquartile range). Categorical variables are expressed as percent (%). After we further stratified subjects according to statin assignments of two guidelines, demographic and biochemical characteristics of these subgroups were compared using one-way analysis of variance (ANOVA) with the Scheffe’s method as post-hoc analysis or the Kruskal-Wallis test with the Dunn procedure for continuous variables or the chi-squared test for categorical variables.

McNemar tests were used to compare the sensitivities and specificities of the two guidelines in determining statin therapy in subjects with subclinical coronary atherosclerosis. The 95% confidence intervals (CIs) were calculated by the Wilson score method without continuity correction [29]. The degree of agreement between the two guidelines on the recommendation of statin therapy was assessed by Cohen’s kappa coefficient, and the classification suggested by Landis and Kock was used to estimate strength of agreement [30]. To compare the two risk assessment methods (i.e., 10 year ASCVD and Framingham risk scoring system) for the detection of subjects with subclinical coronary atherosclerosis, we conducted receiver operating characteristics (ROC) curves and calculated the areas under the curve (AUC). The Youden index was used to identify the best cut-off point. Comparisons of ROC curves were performed by MedCalc® version 11.6.1.0 for Windows (MedCalc Software, Mariakerke, Belgium) according to the method described by DeLong et al [31]. All statistical analyses except ROC curves were performed using SPSS version 19.0 for Windows (SPSS Inc., Chicago, IL). A *P* value of < .05 was considered statistically significant.

## Results

### Clinical and biochemical characteristics of study subjects

Clinical and biochemical characteristics of the 5,837 subjects are shown in Table 1. The mean age was 53.5 ± 7.9 years (range 20–79 years), and 4,209 (72.1%) were men. Among all patients, 432 (7.4%) subjects had significant coronary artery stenosis and 2,330 (39.9%) had coronary plaques. Coronary artery calcification defined as a CACS of 0, 1–100, 101–300, and >300 was observed in 3,881 (66.7%) 1,406 (24.2%), 347 (6.0%), and 185 (3.2%) subjects, respectively.

**Table 1:** Baseline demographic and clinical characteristics of all subjects.

Variables	N = 5,837
**Age (years)**	53.5 ± 7.9
**Age ≥ 40 years (%)**	96.5
**Sex, % male**	72.1
**BMI (kg/m** ^**2**^ **)**	24.6 ± 3.0
**WC (cm)**	85.6 ± 8.4
**Systolic BP (mmHg)**	120.1 ± 13.3
**Diastolic BP (mmHg)**	76.8 ± 10.6
**Current smoker (%)**	23.7
**Moderate drinker (%)**	48.1
**Physically active (%)**	43.8
**Family history of diabetes (%)**	24.3
**Diabetes (n, %)**	750 (12.8)
**Hypertension (n, %)**	1897 (32.5)
**FPG (mg/dL)**	104.1 ± 20.3
**HbA1c (%)**	5.6 (5.3–5.9)
**Total cholesterol (mg/dL)**	197.2 ± 33.2
**TG (mg/dL)**	109 (79–158)
**LDL-C (mg/dL)**	123.3 ± 28.9
**HDL-C (mg/dL)**	53.5 ± 13.7
**Uric acid (mg/dL)**	5.6 ± 1.4
**AST (U/L)**	25 (21–31)
**ALT (U/L)**	22 (16–31)
**GGT (U/L)**	22 (15–37)
**Number of Framingham risk factors**	1 (0–2)
**10-year Framingham risk score (%)**	6 (2–10)
**10-year ASCVD risk score (%)**	4.6 (2.0–8.9)
**Significant stenosis (n, %)**	432 (7.4)
**CACS**	0 (0–9)
**CACS category**	
** 0 (n, %)**	3881 (66.7)
** 1–100 (n, %)**	1406 (24.2)
** 101–300 (n, %)**	347 (6.0)
** >300 (n, %)**	185 (3.2)
**Plaques**	
** Any plaque (n, %)**	2330 (39.9)
** CAP (n, %)**	1535 (26.3)
** NCAP (n, %)**	1047 (17.9)
** MCAP (n, %)**	493 (8.4)

BMI, body mass index; WC, waist circumference; BP, blood pressures; FPG, fasting plasma glucose; HbA1c, hemoglobin A1c; TG, triglyceride; LDL-C, low-density lipoprotein cholesterol; HDL-C, high-density lipoprotein cholesterol; AST, aspartate aminotransferase; ALT, alanine aminotransferase; GGT, gamma-glutamyl transferase; ASCVD, atherosclerotic cardiovascular disease; CACS, coronary artery calcium scores; CAP, calcified plaque; NCAP, noncalcified plaque; MCAP, mixed plaque.

### Assignment to statin according to the 2013 ACC/AHA and 2004 ATP III guidelines

The distribution of subjects who were candidates for statin therapy is shown in Table 2. Statins were assigned to 1,963 (33.6%) subjects according to the 2013 ACC/AHA guidelines and 955 (16.4%) according to 2004 ATP III guidelines.

**Table 2:** Distribution of subjects who were candidates for lipid-lowering drug therapy for primary prevention (age 20–79 years).

	Total	Men	Women
	(n = 5,837)	(n = 4,209)	(n = 1,628)
	N (%)	N (%)	N (%)
**2013 ACC/AHA guideline**			
** LDL-C ≥ 190 mg/dl**	90 (1.5)	46 (1.1)	44 (2.7)
** Diabetes & 40–75 & LDL 70–189 mg/dL**	663 (11.4)	558 (13.3)	105 (6.4)
** No Diabetes & 40–75 & LDL 70–189 mg/dL& ASCVD≥ 7.5%**	1210 (20.7)	1134 (26.9)	76 (4.7)
**Total candidates**	1963 (33.6)	1738 (41.3)	225 (13.8)
**2004 ATP III guideline**			
** CHD risk equivalents** * **& LDL-C≥ 100 mg/dl**	597 (10.2)	510 (12.1)	87 (5.3)
** No Diabetes & CHD risk factor ≥ 2**			
** CHD risk 10–20% & LDL-C ≥ 130 mg/dl**	263 (4.5)	258 (6.1)	5 (0.3)
** CHD risk <10% & LDL-C ≥ 160 mg/dl**	49 (0.8)	35 (0.8)	14 (0.9)
** No CHD & no Diabetes & CHD risk factor 0–1**			
** LDL-C ≥ 190 mg/dl**	46 (0.8)	15 (0.4)	31 (1.9)
**Total candidates**	955 (16.4)	818 (19.4)	137 (8.4)
**Subjects eligible for statins by 2013 ACC/AHA guideline only**	1110 (19.0)	1013 (24.1)	97 (6.0)
**Subjects eligible for statins by 2004 ATP III guideline only**	102 (1.7)	93 (2.2)	9 (0.6)
**Subjects eligible for statins by both guidelines**	853 (14.6)	725 (17.2)	128 (7.9)

ACC/AHA, American College of Cardiology / American Heart Association; LDL-C, low-density lipoprotein cholesterol; ASCVD, atherosclerotic cardiovascular disease; ATP, Adult Treatment Panel; CHD, coronary heart disease.

*Diabetes or CHD risk factor ≥ 2 & CHD risk >20%.

When we analyzed the distribution of subjects who were recommended statin therapy according to these two guidelines, 64.6% (3,772/5,837) of subjects were not recommended statin therapy according to both guidelines. Among those who were recommended statin therapy by either guidelines, 1.7% (102/5,837) was recommend statin therapy only by the 2004 ATP III guidelines, 19.0% (1,110/5,837) only by the 2013 ACC/AHA guidelines, and 14.6% (853/5,837) by both guidelines (Table 2). The level of agreement between these two guidelines on recommending statin therapy was found to be moderate (kappa = 0.451, *P* < .001).

Clinical and biochemical characteristics of each group for whom statins would be assigned/would not be assigned (group 1–4) according to 2004 ATP III and 2013 ACC/AHA guidelines is shown in S1 Table. In post hoc analysis comparing group 2 (assigned statin therapy according to only 2004 ACC/AHA guideline) and group 3 (assigned statin therapy according to only 2013 ACC/AHA guideline), subjects in group 3 were significantly older and showed lower prevalence of hypertension than group 2 (S1 Table). Although subjects in group 3 showed lower levels of total cholesterol, triglyceride, LDL-C, alanine aminotransferase (ALT) and gamma-glutamyl transferase (GGT), they showed significantly higher CACS and higher prevalence of CAP than group 2 (S1 Table).

### Sensitivity and specificity of 2004 ATP III and 2013 ACC/AHA guidelines for statin eligibility according to the subclinical coronary atherosclerosis

**Fig 1:**
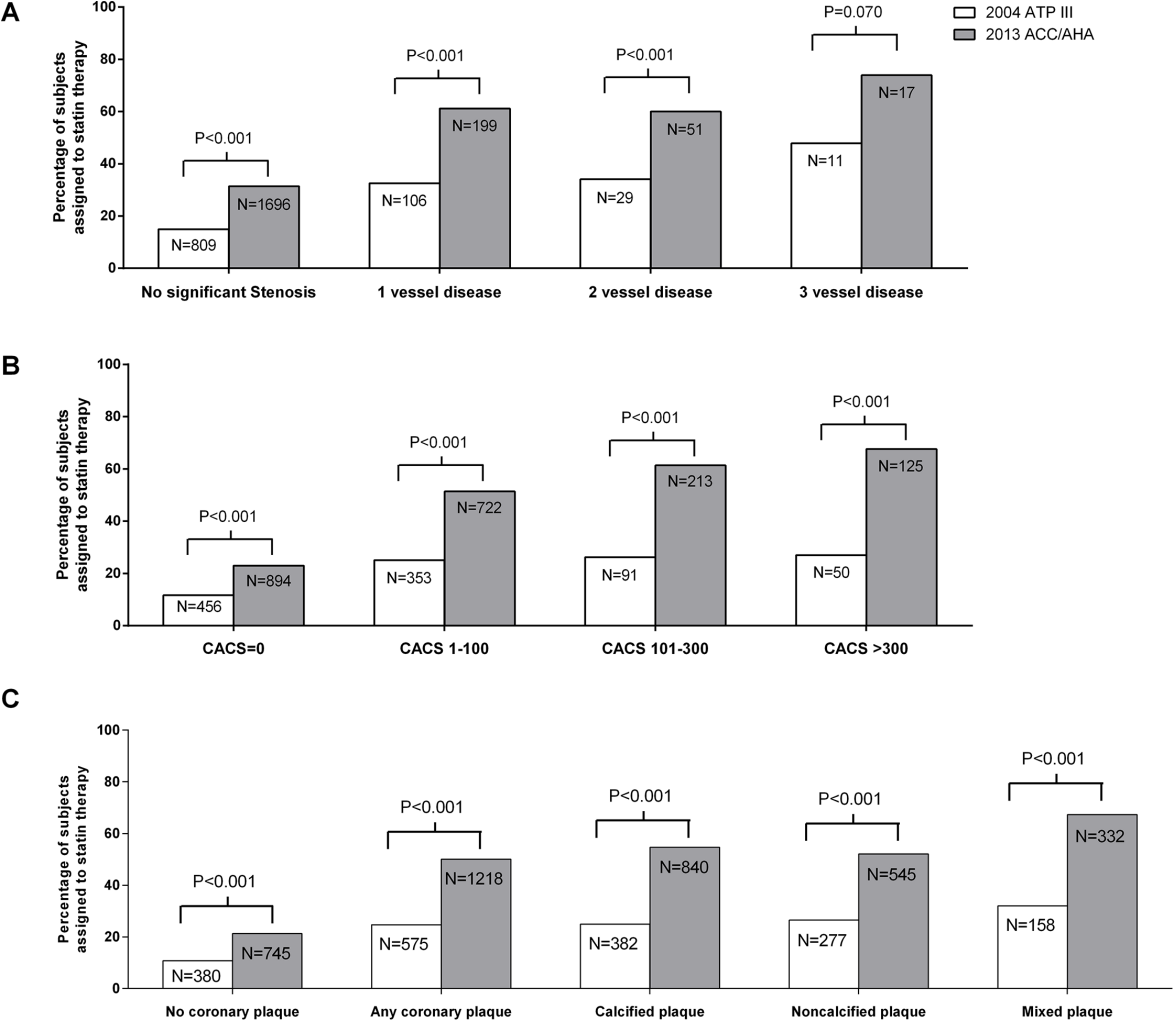
Differences between 2004 ATP III and 2013 ACC/AHA guideline on statin recommendation according to coronary atherosclerosis. Percentage of subjects with or without significant coronary artery stenosis (A), coronary artery calcium score (CACS) of 0, 1–100, 101–300, and >300 (B), coronary plaque (C), who were recommended statin therapy according to 2004 ATP III versus 2013 ACC/AHA guideline.

The difference between the 2004 ATP III and 2013 ACC/AHA guidelines on statin recommendation in subjects with subclinical atherosclerosis is shown in Fig 1. A significantly higher proportion of subjects with significant coronary stenosis (Fig 1A), calcified coronary artery (Fig 1B), or plaques regardless of their subtypes (Fig 1C) was recommended statin therapy using the 2013 ACC/AHA guidelines than using the 2004 ATP III guidelines. When subjects with significant stenosis were further categorized as 1-, 2-, 3-VD, 32.6%, 34.1% and 47.8% of subjects were assigned to statin treatment based on 2004 guidelines, respectively (Fig 1A). In contrast, 63.7%, 63.5%, and 78.3% of subjects, respectively, were assigned statin treatment based on the 2013 ACC/AHA guidelines, which was significantly higher than the rates based on the 2004 ATP III guidelines (Fig 1A). When subjects were categorized according to CACS of 0, 1–100, 101–300, and >300, more subjects in each category were recommended statin therapy according to 2013 ACC/AHA guidelines than to the 2004 ATP III guidelines (CACS of 0; 23.0% vs. 11.7%, CACS of 1–100; 51.4% vs. 25.1%, CACS of 101–300; 61.4% vs. 26.2%, CACS of>300; 67.6% vs. 27.0%, Fig 1B). Similarly, more subjects with no coronary plaque, any coronary plaque, CAP, NCAP, or MCAP were assigned to statin treatment according to 2013 ACC/AHA guidelines than by 2004 ATP III guidelines (no coronary plaque; 21.2% vs. 10.8%, any coronary plaque; 52.3% vs. 24.7%, CAP; 54.7% vs. 24.9%, NCAP; 52.1% vs. 26.5%, MCAP; 67.3% vs. 32.0%, respectively, Fig 1C).

When we compared the diagnostic characteristics of these two guidelines in terms of alignment of statin therapy in subjects with subclinical coronary atherosclerosis, the sensitivity of the 2013 ACC/AHA guidelines was significantly higher than that of the 2004 ATP III guidelines for detecting significant coronary stenosis (61.8% [95% CI, 57.1–66.3] vs. 33.8% [95% CI, 29.5–38.4], respectively, *P* < .001, Table 3). However, the specificity of the 2013 ACC/AHA guidelines was lower than that of the 2004 ATP III guidelines (68.6% [95% CI, 67.4–69.9] vs. 85.0% [95% CI, 84.1–86.0], *P* < .001, Table 3). Similarly, for detecting coronary artery calcification and plaques (regardless of its subtypes), the 2013 ACC/AHA guidelines presented significantly higher sensitivity, but lower specificity compared to the 2004 ATP III guidelines (Table 3).

**Table 3:** Diagnostic characteristics of the 2004 ATP III and 2013 ACC/AHA guidelines in assigning statin therapy in subjects with subclinical coronary atherosclerosis.

MDCT findings	N (%)	2004 ATP III		2013 ACC/AHA		*P* value*	*P* value^†^
		Sensitivity	Specificity	Sensitivity	Specificity		
**Significant stenosis**	432 (7.4%)	33.8 (29.5–38.4)	85.0 (84.1–86.0)	61.8 (57.1–66.3)	68.6 (67.4–69.9)	< .001	< .001
**CACS>0**	1945 (33.4%)	25.5 (23.6–27.5)	88.3 (87.2–89.3)	54.7 (52.5–56.9)	77.0 (75.7–78.3)	< .001	< .001
**CACS>100**	533 (9.2%)	26.5 (22.9–30.4)	84.7 (83.7–85.6)	63.6 (59.4–67.6)	69.4 (68.2–70.7)	< .001	< .001
** Any plaque**	2330 (39.9%)	24.7 (23.0–26.5)	89.2 (88.1–90.2)	52.3 (50.2–54.3)	78.8 (77.4–80.1)	< .001	< .001
** CAP**	1535 (26.3%)	24.9 (22.8–27.1)	86.7 (85.6–87.7)	54.7 (52.2–57.2)	73.9 (72.6–75.2)	< .001	< .001
** NCAP**	1047 (17.9%)	26.5 (23.9–29.2)	85.8 (84.8–86.8)	52.1 (49.0–55.1)	70.4 (69.1–71.7)	< .001	< .001
** MCAP**	493 (8.4%)	32.0 (28.1–36.3)	85.1 (84.1–86.0)	67.3 (63.1–71.3)	69.5 (68.2–70.7)	< .001	< .001

95% Confidence intervals are in brackets. MDCT, multi-detector computed tomography; ATP, Adult Treatment Panel; ACC/AHA, American College of Cardiology / American Heart Association; CACS, coronary artery calcium scores; CAP, calcified plaque; NCAP, noncalcified plaque; MCAP, mixed plaque.

*P value for sensitivity.

^**†**^ *P* value for specificity.

### Comparison of two risk scoring systems for detecting subclinical coronary atherosclerosis

Lastly, to compare the efficacy of the 10-year Framingham and 10-year ASCVD risk scoring systems for assigning subjects with subclinical atherosclerosis to statin therapy, we further excluded subjects that were not between the ages of 40 and 75 years and those with a LDL-C level of ≥190 mg/dL and/or diabetes, in whom the calculation of the 10-year ASCVD risk is not indicated [1]. After these exclusions, we conducted ROC curve analysis and compared the two risk scoring systems for accuracy in detecting subclinical coronary atherosclerosis in the remaining 4,807 subjects (Table 4). The AUCs of the 10-year ASCVD risk scores for detecting significant coronary stenosis, higher CACS, and coronary plaques were significantly higher than those of the 10-year Framingham risk scoring system. The optimal cut-off value for detecting significant stenosis was 5.85% and 6% in the 10-year ASCVD scoring system and the 10-year Framingham risk scoring system, respectively (Table 4).

**Table 4:** Comparison of sensitivity and specificity between Framingham and 10-year ASCVD risk scoring system for alignment of statin therapy in subjects with subclinical coronary atherosclerosis.

MDCT findings	Framingham risk scoring				2013 ASCVD risk scoring				*P* value
	Cut off	Sensitivity	Specificity	AUC (95% CI)	Cut off	Sensitivity	Specificity	AUC (95% CI)	(2004 vs. 2013)
**Significant stenosis**	6	70.6	63.3	0.71 (0.69–0.72)	5.85	70.6	66.8	0.73 (0.72–0.74)	.025
**CACS>0**	5	71.3	61.2	0.72 (0.71–0.73)	4.25	74.4	62.1	0.75 (0.73–0.76)	.027
**CACS>100**	5	78.4	53.5	0.70 (0.69–0.71)	4.86	77.3	59.6	0.74 (0.73–0.76)	.044
**Any plaque**	5	69.3	63.4	0.72 (0.71–0.73)	4.26	71.3	64.1	0.74 (0.73–0.76)	.024
**CAP**	4	79.0	49.7	0.69 (0.68–0.71)	3.85	78.5	54.2	0.73 (0.71–0.74)	.032
**NCAP**	5	70.0	55.3	0.67 (0.66–0.68)	5.16	62.9	63.7	0.68 (0.67–0.69)	.012
**MCAP**	6	71.1	63.7	0.72 (0.71–0.73)	6.47	66.3	71.2	0.74 (0.73–0.75)	.022

ASCVD, atherosclerotic cardiovascular disease; AUC, areas under the curve; CACS, coronary artery calcium scores; CAP, calcified plaque; NCAP, noncalcified plaque; MCAP, mixed plaque.

## Discussion

In this study, there was improved assignment of statin treatment in subjects with subclinical coronary atherosclerosis when the 2013 ACC/AHA guidelines were applied compared to when the 2004 ATP III guidelines were used. Overall, the 2013 ACC/AHA guidelines resulted in better discrimination of subclinical coronary atherosclerosis detected by CCTA including coronary stenosis, higher CACS and coronary plaques than did the 2004 ATP III guidelines in this Korean population.

In previous studies comparing the 2013 ACC/AHA and 2004 ATP III guidelines in detecting subjects with coronary atherosclerosis who underwent CCTA, the new guidelines more accurately assigned statins to patients with coronary atherosclerosis than 2004 ATP III guidelines [15, 16]. Pursnani et al. showed the superiority of the 2013 ACC/AHA guidelines in predicting CHD detected by CCTA in patients who presented to the emergency department with acute chest pain but who were not diagnosed with acute coronary syndrome [15]. In addition, Johnson et al. also reported that the 2013 ACC/AHA guidelines better reflected the severity of atherosclerosis compared to the 2004 ATP III guidelines in patients who underwent CCTA due to various reasons, including atypical chest pain [16]. Most recently, Kim et al. showed that the number of subjects with a CACS of >20 and >100 increased according to increasing 10-year ASCVD risk quartile in a cohort of Korean subjects [32]. However, until now, there were no published data comparing these two guideline systems (i.e., 2013 ACC/AHA versus 2004 ATP III guidelines) in their diagnosis of radiologic abnormalities of coronary artery (e.g., significant stenosis, coronary calcification, and existence of plaque) detected by CCTA in an Asian population. Furthermore, since we included subjects who underwent CCTA during ‘routine health evaluation’, most of them did not have any symptoms of CHD and they showed relatively lower 10 year ASCVD risk score (median 4.6%) compared to the previous studies of Pursnani et al. (median 5.7%) [15] or Johnson et al. (median 7.4%) [16]. Thus, our study seems to be more valuable in evaluating the efficacy of the new guidelines for ‘primary prevention’ compared to previous studies [15, 16].

Currently, growing evidence indicates that CCTA not only accurately detects the presence and extent of CHD [2, 33] but also predicts CVD events [22, 25, 34, 35]. In previous meta-analysis investigating the correlation between abnormal CCTA findings and CVD risk, the risk for all CVD events (cardiovascular death, nonfatal myocardial infarction, unstable angina requiring hospitalization, and revascularization) increased 10.7-fold in subjects with significant coronary artery stenosis and 4.5-fold in subjects with any coronary atherosclerotic plaque [34]. In addition, the risk of CHD increased 7.73-fold in subjects with a CACS of 101–300, and increased 9.67-fold in subjects with a CACS of >300 in a study using data from the Multi-Ethnic Study of Atherosclerosis (MESA) cohort [35]. CACS has been reported to be useful not only for predicting future CVD, but also for selecting asymptomatic subjects who may benefit from polypill treatment (combination of aspirin, a beta-blocker, an angiotensin-converting enzyme inhibitor, and a statin) to reduce CVD events [36]. Considering previous reports that subjects with abnormal CCTA findings have a higher CVD risk [34–36], our results imply that the 2013 ACC/AHA guidelines might be beneficial for the selection of subjects with high CVD risk who may benefit from statin therapy as well as for the detection of abnormal CCTA findings.

In this study, subjects who were eligible for statins only by 2013 ACC/AHA guidelines were significantly older than subjects eligible for statins only by 2004 ATP III guidelines (S1 Table). In addition, they had higher CACS and higher prevalence of CAP, despite their lower prevalence of hypertension and lower levels of conventional cardiovascular risk factors including lower levels of total cholesterol, LDL-C, ALT and GGT (S1 Table). Considering that age is the strongest factor contributing to higher CACS [37, 38], increased number of older adults eligible for statin treatment under the new guidelines might result in higher prevalence of CACS. Our analysis indicated that 2013 ACC/AHA guideline is presumed to be more sensitive in detecting age related progression of coronary atherosclerosis. Further studies are needed to validate this speculation.

Of note, in our study, the calculated optimal cut-off values for the 10-year ASCVD scoring system for detecting significant stenosis, CACS >100, and any plaque were 5.85%, 4.86%, and 4.26%, which were lower than the recommended value of 7.5% [1]. According to the 2013 ACC/AHA guidelines, statin treatment was regarded to be beneficial for reducing CVD event in subjects with a 10-year ASCVD risk ranging between 5 and 7.5% [1]. However, high intensity statin therapy was not generally recommended in this group because the potential adverse effects may outweigh the potential benefit [1]. Although our study did not directly compare the risk and benefit of statin treatment for reducing CVD events, the relatively low cut-off values in our analysis indicate that a cut-off value of 7.5% suggested by the 10-year ASCVD scoring system might not be too low to detect coronary atherosclerosis in Asian population.

In our analysis, the sensitivity of the 2013 ACC/AHA guidelines was significantly higher but the specificity was lower for detecting various abnormalities on CCTA findings compared to the 2004 ATP III guidelines (Table 3), which was in line with previous reports [15, 16]. Regarding specificity, more subjects with a validated ‘normal CCTA finding’ (i.e., CACS of 0 and no coronary plaque) [25] were recommended statin treatment by the 2013 ACC/AHA guidelines than by the 2004 ATP III guidelines (20.8% versus 10.7%, *P* < .001, data not shown). Because subjects with ‘normal CCTA findings’ were reported to have a very low risk of future CVD events [4, 22, 25, 36], this result raises concern of statin over-treatment according to the 2013 ACC/AHA guidelines. However, considering the findings that only 47.8% of subjects with 3-VD and 27.0% of subjects with CACS of >300 were recommended statin treatment for primary prevention based on the 2004 ATP III guidelines, while 73.9% and 67.6% were recommended statin therapy according to the 2013 ACC/AHA guidelines (Fig 1), the new guidelines appear to be valuable in assigning statin treatment to high-risk patients.

Our current analyses had several limitations that should be noted. First, participants were voluntarily recruited during routine health examinations, which may have led to selection bias; thus, our results may not be representative of the general Korean population. Second, in our study, there was no surveillance of peripheral artery disease, abdominal aortic aneurysm, and carotid artery disease. As the definition of ‘clinical ASCVD’ according to the 2013 ACC/AHA guidelines included peripheral artery disease as well as stroke and CHD, subjects with peripheral artery disease may have been incorrectly included in our analysis [1]. In addition, as ‘CHD risk equivalents’ in high risk category included subjects with peripheral artery disease, abdominal aortic aneurysm, or carotid artery disease according to the 2004 ATP III guidelines, they might not be classified as high risk group in our analysis [2]. Lastly, although our results showed the superiority of the 2013 ACC/AHA guidelines over the 2004 ATP III guidelines for detecting subclinical coronary atherosclerosis, the absolute benefit or risk of statin treatment according to these guidelines remains unclear in this population because we did not investigate the actual occurrence of CVD or statin-related adverse events.

Despite these limitations, our study has robust features, in that we included a large number of asymptomatic subjects who underwent CCTA. In particular, we firstly showed that even in an Asian population, the 2013 ACC/AHA guidelines were more sensitive in identifying subjects with subclinical atherosclerosis than previous guidelines. Further randomized trials that evaluate the reduction in CVD by statin treatment according to the two guidelines are needed to compare the actual benefit in statin assignment in an Asian population.

## Supporting Information

S1 Table Clinical characteristics of subjects for whom statins would be assigned/would not be assigned according to 2004 ATP III and 2013 ACC/AHA guideline. (DOCX)

## Abbreviations

ACC/AHA: American College of Cardiology / American Heart Association
ALT: alanine aminotransferase
ASCVD: atherosclerotic cardiovascular disease
AST: aspartate aminotransferase
ATP: Adult Treatment Panel
AUC: areas under the curve
BMI: body mass index
BP: blood pressures
CACS: coronary artery calcium scores
CAP: calcified plaque
CCTA: coronary computed tomography angiography
CHD: coronary heart disease
CVD: cardiovascular disease
FPG: fasting plasma glucose
GGT: gamma-glutamyl transferase
HbA1c: hemoglobin A1c
HDL-C: high-density lipoprotein cholesterol
LDL-C: low-density lipoprotein cholesterol
MCAP: mixed plaque
MDCT: multi-detector computed tomography
MESA: Multi-Ethnic Study of Atherosclerosis
NCAP: noncalcified plaque
NCEP: National Cholesterol Education Program
ROC: receiver operating characteristics
TG: triglyceride
VD: vessel disease
WC: waist circumference.
