# Supplementary material for: 2013 ACC/AHA versus 2004 NECP ATP III Guidelines in the Assignment of Statin Treatment in a Korean Population with Subclinical Coronary Atherosclerosis
Source: PLoS One. 2015 Sep 15;10(9):e0137478. doi: 10.1371/journal.pone.0137478 (PMC4570667; doi:10.1371/journal.pone.0137478)
Supplement: S1 Table — (DOCX) [file pone.0137478.s001.docx]

| **Variables** | **Group 1**  **Not eligible for statins by both guidelines**  **N=3772** | **Group 2**  **Eligible for statins by ATP III only**  **N=102** | **Group 3**  **Eligible for statins by ACC/AHA only**  **N=1110** | **Group 4**  **Eligible for statins by both guidelines**  **N=853** | **P value** | **P value between group 2&3^*^** |
| --- | --- | --- | --- | --- | --- | --- |
| **Age (years)** | 51.0 ± 6.7 | 52.5 ± 10.7 | 60.5 ± 7.1 | 55.3 ± 7.4 | <.001 | <.001 |
| **Sex, % male** | 63.0% | 91.2% | 91.3% | 85.0% | <.001 | .977 |
| **BMI (kg/m^2^)** | 24.2 ± 2.9 | 25.6 ± 2.9 | 25.0 ± 2.9 | 25.5 ± 2.8 | <.001 | .259 |
| **WC (cm)** | 84.0 ± 8.3 | 89.0 ± 7.8 | 88.3 ± 7.4 | 89.0 ± 7.7 | <.001 | .882 |
| **Systolic BP (mmHg)** | 117.4 ± 12.4 | 124.5 ± 14.8 | 124.6 ± 12.9 | 125.6 ± 14.2 | <.001 | .999 |
| **Diastolic BP (mmHg)** | 75.1 ± 10.3 | 81.5 ± 11.6 | 79.5 ± 9.6 | 80.6 ± 10.9 | <.001 | .342 |
| **Current smoker (%)** | 15.6% | 35.3% | 37.8% | 47.3% | <.001 | .797 |
| **Moderate drinker (%)** | 46.0% | 61.4% | 53.4% | 53.6% | <.001 | .182 |
| **Diabetes (%)** | 1.4% | 14.7% | 15.0% | 60.4% | <.001 | .927 |
| **Hypertension (%)** | 23.0% | 61.8% | 47.9% | 51.1% | <.001 | .007 |
| **FPG (mg/dL)** | 98.77 ±11.7 | 107.2 ± 25.2 | 105.7 ± 19.9 | 125.1 ± 32.5 | <.001 | .875 |
| **HbA1c (%)** | 5.5 (5.2-5.7) | 5.7 (5.5-6.0) | 5.7 (5.4-6.0) | 6.2 (5.7-6.9) | <.001 | .320 |
| **Total cholesterol (mg/dL)** | 193.9 ± 30.7 | 231.2 ± 33.1 | 188.1 ± 28.5 | 219.7 ± 37.0 | <.001 | <.001 |
| **TG (mg/dL)** | 99.0 (72.0-141.0) | 141.0 (111.8-184.8) | 122.0 (87.0-170.3) | 144.0 (105.0-196.0) | <.001 | <.001 |
| **LDL-C (mg/dL)** | 119.4 ± 26.7 | 155.4 ± 25.6 | 116.7 ± 23.9 | 145.8 ± 31.1 | <.001 | <.001 |
| **HDL-C (mg/dL)** | 56.1 ± 13.9 | 47.9 ± 12.1 | 49.1 ± 12.4 | 48.3 ± 11.3 | <.001 | .861 |
| **Uric acid (µmol/L)** | 5.5 ± 1.4 | 6.3 ± 1.4 | 5.9 ± 1.4 | 5.8 ± 1.3 | <.001 | .174 |
| **AST (U/L)** | 24 (21-30) | 26 (22-32) | 26 (22-31) | 27 (22-34) | <.001 | .67 |
| **ALT (U/L)** | 21 (16-29) | 26 (19-35) | 22 (17-31) | 26 (19-37) | <.001 | .010 |
| **GGT (U/L)** | 19 (13-33) | 31 (20-49) | 25 (17-38) | 31 (20-48) | <.001 | .010 |
| **10-year Framingham risk score (%)** | 4 (1-6) | 10 (6-16) | 12 (8-12) | 12 (6-16) | <.001 | .190 |
| **10-year ASCVD risk score (%)** | 2.9 (1.3-4.7) | 6.5 (5.0-7.3) | 10.5 (8.5-13.9) | 12.1 (8.3-17.4) | <.001 | <.001 |
| **Significant stenosis (%)** | 146 (3.9%) | 19 (18.6 %) | 140 (12.6 %) | 127(14.9%) | <.001 | .085 |
| **CACS** | 0 (0-0) | 0 (0-22.9) | 3.3 (0-54.0) | 2 (0-45.9) | <.001 | .010 |
| **CACS category** |  |  |  |  | <.001 | .022 |
| **0 (%)** | 2927 (77.8%) | 60 (58.8%) | 498 (45.0%) | 396 (46.7%) |  |  |
| **1-100 (%)** | 653 (17.4%) | 31 (30.4%) | 400 (36.2%) | 322 (38.0%) |  |  |
| **101- 300 (%)** | 130 (3.5%) | 4 (3.9%) | 126 (11.4%) | 87 (10.3%) |  |  |
| **>300 (%)** | 53 (1.4%) | 7 (6.9%) | 82 (7.4 %) | 43 (5.1%) |  |  |
| **Any plaque (%)** | 1058 (28.0%) | 54 (52.9%) | 697(62.8%) | 521 (61.1%) | <.001 | .050 |
| **CAP (%)** | 664 (17.6%) | 31 (30.4%) | 489 (44.1%) | 351 (41.1%) | <.001 | .008 |
| **NCAP (%)** | 472 (12.5%) | 30 (29.4%) | 298 (26.8%) | 247 (29.0%) | <.001 | .577 |
| **MCAP (%)** | 149 (4.0%) | 12 (11.8%) | 186 (16.8%) | 146 (17.1%) | <.001 | .192 |

**S1 Table. Clinical characteristics of subjects for whom statins would be assigned/would not be assigned according to 2004 ATP III and 2013 ACC/AHA guideline**

**^*^**To compare group 2 and 3, post hoc analysis using one-way analysis of variance (ANOVA) with the Scheffe’s method or Kruskal-Wallis test with the Dunn procedure was performed.
